# Comparative Analysis of the Amount of Plaque Formation and Associated Gingival Inflammation in Deciduous, Mixed and Permanent Dentition

**DOI:** 10.5005/jp-journals-10005-1014

**Published:** 2009-12-26

**Authors:** Abhay Agarwal, Usha Rehani, Vivek Adlakha, Mayur Kaushik, Noopur Kaushik

**Affiliations:** 1 Reader, Department of Pedodontics, Subharti Dental College, Meerut, Uttar Pradesh, India; 2 Professor and Head, Department of Pedodontics, Subharti Dental College, Meerut, Uttar Pradesh, India; 3 Senior Lecturer, Department of Pedodontics, Subharti Dental College, Meerut, Uttar Pradesh, India; 4 Reader, Department of Periodontics, Subharti Dental College, Meerut, Uttar Pradesh, India; 5 Postgraduate Student, Department of Pedodontics, Subharti Dental College, Meerut, Uttar Pradesh, India

**Keywords:** Gingivitis, plaque, deciduous, mixed, permanent dentition.

## Abstract

Periodontal diseases in children and adolescents comprise mainly of gingivitis. Gingivitis is an inflammation involving the gingival tissues next to the teeth. Marginal gingivitis is the most common form of periodontal disease and starts in early childhood. However, severe gingivitis is relatively uncommon in children, although a large population has a mild, reversible type of gingivitis. The major etiologic factors associated with gingivitis and more significantly periodontal diseases are uncalcified and calcified bacterial plaque.

The gingival tissues in children are different from those in adults. Due to these differences in the structure between the gingival tissues of the child and the adult, even the clinical features and disease manifestations differ according to the age of an individual. It has been observed in earlier studies that children with a deciduous dentition seem to respond to plaque formation with less gingivitis than adults with a permanent dentition.

Thus, in this study, the occurrence of gingivitis in response to plaque was studied and compared in the deciduous, mixed and permanent dentitions.

## INTRODUCTION

Periodontal diseases in children and adolescents comprise mainly of gingivitis. Gingivitis is an inflammation involving the gingival tissues next to the teeth. Microscopically, it is characterized by the presence of an inflammatory exudate, edema, some destruction of collagenous gingival fibers and ulceration and proliferation of the epithelium facing the tooth and the gingiva attached to it. It has been seen that *marginal gingivitis is the most common form of periodontal disease* and starts in early childhood.^1^

Severe gingivitis is relatively uncommon in children, although it has been shown that a large population has a mild, reversible type of gingivitis.^1^ The major etiologic factors associated with gingivitis and more significantly periodontal diseases are uncalcified and calcified bacterial plaque. Dental plaque has been defined as "a specific but highly variable structural entity, resulting from the sequential colonization of microorganisms on tooth surfaces, restorations and other parts of the oral cavity. It is composed of salivary components like mucin, desquamated epithelial cells, debris and microorganisms, all embedded in an extracellular gelatinous matrix." - WHO 1961.

The relationship between plaque and gingivitis was first established by Loe et al^2^ in his landmark study: *The Classical Gingivitis study*. Loe had observed that gingivitis developed in individuals after 7-21 days in the absence of personal plaque removal, thus establishing the etiologic role of plaque in the causation of gingival inflammation.

Longitudinal studies, as well as cross-sectional studies of children of different ages, have shown that the severity and prevalence of gingivitis increases with age.^3^-^6^ Since the amount of plaque was not recorded in these studies, one cannot tell whether the age-related differences in gingival inflammation are a consequence of intrinsic factors, or whether they simply reflect differences in the amount of plaque, caused for instance by age-related variations in oral hygiene habits.

Thus, the present pilot study was conducted to monitor de novo plaque formation and associated alterations of the gingival conditions in the deciduous, mixed and permanent dentitions of children of different age groups in the Indian population.

## MATERIAL AND METHODS

Thirty children were enrolled in the study from the patients attending the out patient clinic of Department of Pedodontics and Preventive Dentistry at Subharti Dental College, Meerut. They were divided into 3 groups of 10 patients each:

**Group A:** Subjects in the age group of 4-6 years (*deciduous dentition*).

**Group B:** Subjects in the age group of 8-10 years (*mixed dentition*).

**Group C:** Subjects in the age group of 12-14 years (*permanent dentition*).

After a screening examination, each participant was subjected to professional tooth cleaning. Each subject was asked to abstain from all mechanical oral hygiene measures. After one week (day 7), oral examination was performed. This examination included assessments of plaque and gingivitis. The same examinations were repeated at the end of 3 weeks (21 days).

A written consent was taken from the parents of the children who were enrolled in the study. Since gingivitis is a completely reversible disease,^2^ it was assumed that no permanent damage would occur to the periodontium of the children.

The amount of dental plaque was assessed using plaque index of Silness and Loe.^7^ The index was taken at 0, 7 and 21 days. The amount of gingival inflammation was assessed using the gingival index of Loe and Silness.^8^ This index was also taken at 0, 7 and 21 days. Both the indices were taken in the selected index teeth at the four points around each tooth: Mesiobuccal, midbuccal, distobuccal and midpalatal.

The mean scores for the plaque and the gingival index were calculated for each group and the standard deviation was measured. One-way analysis of variance (ANOVA) was then applied to analyze the statistical difference between the 3 groups for both the parameters, i.e. gingival and plaque index. The results were further analyzed using unpaired t-test to compare between all possible combinations of the 3 groups.

## RESULTS

The results are presented in the following tables. Table 1 shows the scores of the plaque index for the 3 groups – Deciduous, mixed and permanent dentition, while Table 2 shows the gingival index scores for the 3 groups.

**Table:** TABLE 1: Plaque index scores

*Patient no.*		*Group A*		*Group B*		*Group C*	
1			2.2		2.6		1.6
2			2		2.5		2
3			2.2		2.7		2.2
4			2.4		2.6		2
5			2.4		2.8		2.4
6			1.8		2.8		1.6
7			2		2.4		2.4
8			2		2.6		2.2
9			2.2		2.6		1.8
10			2.4		2.5		1.8
*Mean*			2.16		2.61		2.01
*SD*			*0.206559*		*0.128668*		*0.298142*

**Table:** TABLE 2: Gingival index scores

*Patient no.*		*Group A*		*Group B*		*Group C*	
1			0.7		1.6		2.4
2			0.6		1.4		2.5
3			0.5		1.5		2.9
4			0.4		1.9		2.8
5			1.1		1.8		2.9
6			0.5		1.8		2.9
7			0.7		1.5		2.8
8			0.3		1.6		2.7
9			0.3		1.7		2.8
10			0.5		1.7		2.9
*Mean*			0.56		1.65		2.76
*SD*			*0.236643*		*0.158114*		*0.177639*

On comparing the plaque index scores for the 3 groups, it was found that plaque index scores were highest for the mixed dentition group (Group B), then the deciduous group (Group A) and least for the permanent dentition group (Group C). On applying one way analysis of variance (ANOVA) the difference between the groups was found to be statistically significant (p < 0.05). Further, when unpaired t-test was applied between all possible combinations of the 3 groups, the results were still statistically significant.

On comparing the gingival index scores for the 3 groups, it was found that gingival index scores were highest for the permanent dentition group (Group C), then the mixed group (Group C) and least for the deciduous dentition group (Group A). On applying one way analysis of variance (ANOVA) the difference between the groups was found to be statistically significant (p < 0.05). Further, when unpaired t-test was applied between all possible combinations of the 3 groups, the results were still statistically significant.

## DISCUSSION

Previous studies have shown that the tendency to develop gingivitis is low among preschool children as compared with young adults.^4^,^5^ The results of the present study are in accordance with these studies. The present results show that at the same level of plaque accumulation, the gingival reaction was lower in the children aged 4-6 years than in older children. In all plaque index classes, reactivity to bacterial irritation was highest in the 12-14 year olds. The reaction in the 8-10 year old children was less intense. Thus, the results suggest that in childhood, there is no clear cut age at which the gingival reaction to bacterial irritation becomes similar to the level seen in adults. Instead gingival reactivity seems to increase gradually from early childhood to adult age.

An experimental gingivitis approach^2^ was used in the present study. This approach affords better possibilities of controlling interfering factors than a cross-sectional model (i.e. measurement of gingival and plaque indexes at a random point of time without telling the patients not to brush for 3 weeks). However, a cross-sectional might also be useful considering that it may be only the early development of gingivitis which is retarded in young children compared to adults and the difference becomes pronounced at a more established stage of the disease, when tissues have been subjected to plaque irritation for a longer period of time.

The reasons for age-related difference in gingival inflammatory reactivity are not known, but differences in both bacteriologic inflammatory cell response between children and adults have been noted. Certain microorganisms commonly associated with periodontitis in adults and considered to be potential periodontal pathogens are not regularly present in children with normal gingiva.^9^,^10^ A difference in subgingival plaque formation between juvenile and adults dogs during a 3 weeks period of plaque development has also been noted.^11^ In the adults dogs, subgingival plaque was frequently present during the experimental period, while in the juvenile dogs such plaque was not observed.

Longhurst et al^11^ reported markedly fewer plasma cells in inflamed gingival tissue of young children than young adults. A similar difference has been found between juvenile and adult dogs during a period of developing experimental gingivitis.^12^ These studies may suggest a limited involvement of the humoral immune system in gingival lesions in young individuals.

In a histologic study by Matsson et al,^12^ a morphologic difference in the dentogingival region between juvenile and adult dogs was also noted. The junctional epithelium in the juvenile dogs with a deciduous dentition was thicker and had a dental cuticle than that of adult dogs with a permanent dentition, which hypothetically may lead to a lower permeability of bacterial products through the gingival tissues of juvenile dogs. However, it is not known whether these morphologic differences are also present in humans, as the junctional epithelium of healthy gingiva around deciduous teeth has not been sufficiently investigated.

There are several possible explanations for the clinically observed difference between children and adults in their tendency to develop gingivitis. Further research into bacteriologic, immunologic and local histologic differences between young and grown-up individuals is needed.
